# Comparison of All-Cause Mortality Rates and Inequities Between Black and White Populations Across the 30 Most Populous US Cities

**DOI:** 10.1001/jamanetworkopen.2020.32086

**Published:** 2021-01-20

**Authors:** Maureen R. Benjamins, Abigail Silva, Nazia S. Saiyed, Fernando G. De Maio

**Affiliations:** 1 Sinai Urban Health Institute, Chicago, Illinois; 2 Loyola University, Chicago, Illinois; 3 DePaul University, Chicago, Illinois; 4 American Medical Association, Chicago, Illinois

## Abstract

**Importance:**

To address elevated mortality rates and historically entrenched racial inequities in mortality rates, the United States needs targeted efforts at all levels of government. However, few or no all-cause mortality data are available at the local level to motivate and guide city-level actions for health equity within the country’s biggest cities.

**Objectives:**

To provide city-level data on all-cause mortality rates and racial inequities within cities and to determine whether these measures changed during the past decade.

**Design, Setting, and Participants:**

This cross-sectional study used mortality data from the National Vital Statistics System and American Community Survey population estimates to calculate city-level mortality rates for the non-Hispanic Black (Black) population, non-Hispanic White (White) population, and total population from January 2016 to December 2018. Changes from January 2009 to December 2018 were examined with joinpoint regression. Data were analyzed for the United States and the 30 most populous US cities. Data analysis was conducted from February to November 2020.

**Exposure:**

City of residence.

**Main Outcomes and Measures:**

Total population and race-specific age-standardized mortality rates using 3-year averages, mortality rate ratios between Black and White populations, excess Black deaths, and annual average percentage change in mortality rates and rate ratios.

**Results:**

The study included 26 295 827 death records. In 2016 to 2018, all-cause mortality rates ranged from 537 per 100 000 population in San Francisco to 1342 per 100 000 in Las Vegas compared with the overall US rate of 759 per 100 000. The all-cause mortality rate among Black populations was 24% higher than among White populations nationally (rate ratio, 1.236; 95% CI, 1.233 to 1.238), resulting in 74 402 excess Black deaths annually. At the city level, this ranged from 6 excess Black deaths in El Paso to 3804 excess Black deaths every year in Chicago. The US rate remained constant during the study period (average annual percentage change, −0.10%; 95% CI, −0.34% to 0.14%; *P* = .42). The racial inequities in rates for the US decreased between 2008 and 2019 (annual average percentage change, −0.51%; 95% CI, −0.92% to −0.09%; *P* =0.02). Only 14 of 30 cities (46.7%) experienced improvements in overall mortality rates during the past decade. Racial inequities increased in more cities (6 [20.0%]) than in which it decreased (2 [6.7%]).

**Conclusions and Relevance:**

In this study, mortality rates and inequities between Black and White populations varied substantially among the largest US cities. City leaders and other health advocates can use these types of local data on the burden of death and health inequities in their jurisdictions to increase awareness and advocacy related to racial health inequities, to guide the allocation of local resources, to monitor trends over time, and to highlight effective population health strategies.

## Key Points

**Question:**

How do all-cause mortality rates and racial inequities in rates vary across the 30 most populous US cities?

**Findings:**

In this cross-sectional study of more than 26 million death records during a 10-year period, city mortality rates differed widely as did inequities between Black and White populations. Overall mortality rates improved in less than half of the 30 cities, and racial inequities worsened in more cities than in which they improved.

**Meaning:**

Given the substantial variation in city-level mortality rates and racial inequities, cities need data specific to their jurisdiction to inform local health policy.

## Teaser

This cross-sectional study examines city-level data from the 30 most populous US cities regarding all-cause mortality rates and racial inequities within cities and seeks to determine whether these measures changed during the past decade.

## Introduction

All-cause mortality, a primary measure of a population’s health, has been documented in the United States since the 1800s. National mortality rates have declined significantly during the past century, until a recent increase in 2015.^1,2^ Although these data are important for benchmarking our nation’s progress, the examination of mortality rates within population subgroups and use of more local data can reveal important differences.^3,4,5,6^

In the United States, racial inequities in all-cause mortality are prominent. Black individuals have had higher death rates than White individuals for as long as records of race-specific mortality have existed.^7^ Although these disparities have generally narrowed, they remain a critical marker of continued injustice.^1,8,9^ However, little is known about racial inequities in mortality at a more local level.

All-cause mortality rates have been shown to vary by region, state, and county.^3,10,11^ For example, in 2016 the age-adjusted all-cause mortality rate (per 100 000 population) at the state level ranged from 492 in California to 768 in Mississippi.^11^ Huge variations at the county level are also observed.^3,10^ However, even counties are large enough to obscure important geographic differences.^12^ Thus, researchers have increasingly focused on obtaining more local data to identify inequities and drive place-based initiatives.^5,13^ Indeed, a recent call to public health action included a demand for “timely, reliable, granular-level (ie, subcounty), and actionable data.”^14^

Cities represent an ideal level of analysis because they correspond to primary political jurisdictions, unlike neighborhoods or census tracts. City officials, public health professionals, and other health advocates need data for their jurisdictions to make evidence-based changes in policies, services, and funding.^14^ In particular, local departments of public health, in partnership with city agencies and offices, create many health-related policies and guide large budgets.^15^ Local health departments serving areas of 500 000 to 999 999 residents spend a mean of $47 million annually, whereas those serving areas with greater than 1 million residents spend a mean of $174 million.^15^

Despite this need for local data, to our knowledge no existing sources provide all-cause mortality rates for the most populous US cities. Several important initiatives, including the 500 Cities project^16^ and the City Health Dashboard,^17^ make city-level health data available; however, neither includes all-cause mortality. The Big Cities Health Inventory does include all-cause mortality, but as of this writing, the latest data were available for only 5 cities.^18^ Furthermore, no city-level information on racial inequities in all-cause mortality was found in any source. It is critical to examine explicit measures of inequities and how these inequities change over time to assess progress in achieving health equity, a fundamental goal of the US Healthy People initiative.^19^

To address these gaps in knowledge, the current study assessed total and race-specific all-cause mortality and inequities between Black and White populations for the 30 most populous US cities. We also examined trends during the past decade to identify cities that have experienced improvements in rates and health equity.

## Methods

This study was reviewed by the Mount Sinai Hospital institutional review board and was ruled exempt from review because it uses publicly available, deidentified data. This study followed the Strengthening the Reporting of Observational Studies in Epidemiology (STROBE) reporting guideline.

### Study Population

We identified the 30 most populous cities through 2013 US Census Bureau data. Inclusion was limited to these cities to ensure enough deaths for examination of Black and White populations for all-cause mortality and the leading causes of death in the broader research project.^20^ The cities, which make up 12.5% of the US population, are listed in eTable 1 in the Supplement along with selected demographic data.

### Data Sources

#### Mortality Data

Mortality data came from the Multiple Cause of Death data files from the National Vital Statistics System.^21^ For 2009 to 2018, we extracted race-specific deaths by age group (ie, 0-4, 5-14, 15-24, 25-34, 35-44, 45-54, 55-64, 65-74, 75-84, and 85 years and older), race and ethnicity, and place of residence. Age groups were selected to closely match those used by the National Vital Statistics System for age adjustment, with an alteration made to combine individuals younger than 1 year and those aged 1 to 4 years into 1 group to match the population data.^1^ We excluded the records of non-US residents and records in which age was missing. Death certificate data were filled out by proxy (eg, funeral director, attending physician).^22^

#### Population Data

For each city, total, race-specific, and age-specific population-based denominators were obtained from the US Census Bureau American Community Survey 5-year population estimates. When calculating rates for a single year, we used the survey’s 5-year estimate for the corresponding year because it provides more reliable estimates than 1-year samples; when calculating outcome measures for a group of 3 years, we used the survey’s 5-year estimate for the middle year and applied a multiplier of 3 to estimate the population during the entire period. Race and ethnicity data in the American Community Survey and census were self-reported.

The total and non-Hispanic White population estimates were drawn directly from the American Community Survey. Although non-Hispanic Black populations are reported in the census, the American Community Survey only provides estimates of the total (Hispanic and non-Hispanic) Black population. We therefore calculated the age-specific proportion of the 2010 Black population that was non-Hispanic and applied this proportion to the Black population data from the American Community Survey to estimate the non-Hispanic Black population. The total city outcomes included all race/ethnicity groups (not just Black and White). County data were used in 3 instances in which city and county governments created a consolidated city (ie, Louisville and Jefferson County, Kentucky; Nashville and Davidson County, Tennessee; and Indianapolis and Marion County, Indiana). Black and White population estimates were subtracted from the total population estimates to find the other population estimates. We summed population data from each of the 30 cities to calculate 30-city combined mortality rates. We subtracted the 30-city combined population from the US population to find the US population minus these 30 cities.

### Statistical Analysis

Age-adjusted total and race-specific mortality rates were calculated for all 30 cities. Age-adjusted rates per 100 000 population were calculated with the standard US population in 2000.^23^ A 3-year average mortality rate was used (2016-2018) to provide a more stable time estimate for the most recent period. One-year rates were used to estimate the average annual percentage change.

Relative inequities were assessed with rate ratios between Black and White populations. We also calculated the number of excess Black deaths by multiplying the age-specific White mortality rates by the corresponding Black populations in each age category. The sum of these products was the number of Black deaths that would be expected if death rates among the White population were applied to this population. We then subtracted the expected deaths from the number of observed deaths to obtain the excess number of deaths annually. We used White individuals as the reference group because they are the largest racial group in the United States and generally have more favorable health outcomes than other racial and ethnic groups.^24^ For rate ratios, we calculated standard errors, and we calculated CIs with a Taylor series expansion technique.^25^

Trends were examined with log linear joinpoint regression models to calculate the average annual percentage changes and their 95% CIs.^26,27^ To assess inequities, we imported the annual log-transformed rate ratios and their standard errors to calculate the average annual percentage changes. The latter are the weighted average of the annual percentage change from the joinpoint model in which the weights equal the length of the annual percentage change interval. This approach provides a more stable estimate of the trend within a fixed interval.^26^ The average annual percentage change helps determine the direction, magnitude, and significance of changes in rates and rate ratios over time. An increase is denoted by an average annual percentage change greater than 0 (*P* < .05) and a decrease, by an average annual percentage change less than 0 (*P* < .05); otherwise, the trend is considered stable. All statistical tests were 2 sided. We used Joinpoint version 4.8.0.1 (National Cancer Institute) to run the joinpoint regression models.

## Results

A total of 26 348 491 death records were assessed for eligibility, and we excluded 51 159 records of non-US residents and 1505 records in which age was missing. Thus, there were 26 295 827 death records from 2009 to 2018 included in the analysis.

### Total Mortality Rates

In 2016 to 2018, the all-cause mortality rate for the US was 759 per 100 000 individuals (Table). The annual US mortality rate did not significantly change between 2009 and 2018 (average annual percentage change, –0.10%; 95% CI, –0.34% to 0.14%; *P* = .42). City rates ranged from 537 per 100 000 individuals (San Francisco) to 1342 per 100 000 individuals (Las Vegas). The combined rate of the 30 cities (724 per 100 000 individuals) was substantially lower than that of the US as a whole. Full data for the 3 periods (2010-2012, 2013-2015, and 2016-2018) are available in eTable 2 in the Supplement.

**Table.** All-Cause Mortality Rates and Measures of Inequities for the United States and 30 Most Populous Cities, 2016 to 2018

Location	Mortality rate, per 100 000 individuals				Rate ratio between Black and White individuals (95% CI)
	Total	Non-Hispanic		Other^a^	
		Black	White		
United States	759	960	777	539	1.236 (1.233-1.238)
Las Vegas, NV	1342	1718	1462	924	1.18 (1.13-1.22)
Baltimore, MD	993	1107	870	343	1.27 (1.23-1.31)
Detroit, MI	984	1048	795	670	1.32 (1.26-1.38)
Memphis, TN	951	1086	786	475	1.38 (1.34-1.43)
Indianapolis, IN^b^	911	1068	896	451	1.19 (1.16-1.23)
Louisville, KY^b^	910	1069	902	425	1.19 (1.15-1.23)
Houston, TX	895	1226	850	734	1.44 (1.41-1.47)
Jacksonville, FL	894	985	926	499	1.06 (1.03-1.09)
Philadelphia, PA	870	1013	827	597	1.22 (1.20-1.25)
Nashville, TN^b^	868	1041	849	470	1.23 (1.18-1.27)
San Antonio, TX	866	1105	918	802	1.20 (1.16-1.25)
Columbus, OH	858	975	870	323	1.12 (1.08-1.16)
Portland, OR	837	1209	849	618	1.42 (1.34-1.52)
Fort Worth, TX	823	1103	831	588	1.33 (1.28-1.38)
Oklahoma City, OK	813	1114	811	535	1.37 (1.31-1.44)
Dallas, TX	795	1092	736	590	1.48 (1.44-1.52)
Chicago, IL	756	1065	644	523	1.65 (1.62-1.68)
El Paso, TX	735	894	847	704	1.05 (0.95-1.18)
Washington, DC	733	993	428	402	2.32 (2.22-2.42)
Charlotte, NC	729	931	678	385	1.37 (1.33-1.42)
Denver, CO	715	918	689	683	1.33 (1.27-1.40)
Austin, TX	687	980	698	563	1.40 (1.33-1.48)
Phoenix, AZ	686	938	694	612	1.35 (1.29-1.42)
Boston, MA	632	735	666	450	1.10 (1.06-1.15)
Los Angeles, CA	619	1102	605	522	1.82 (1.78-1.86)
San Diego, CA	592	857	622	499	1.38 (1.32-1.45)
Seattle, WA	588	945	587	484	1.61 (1.52-1.71)
New York, NY	570	718	542	504	1.33 (1.31-1.34)
San Jose, CA	549	856	654	455	1.31 (1.20-1.42)
San Francisco, CA	537	1102	573	455	1.92 (1.83-2.03)
30 cities combined	724	971	717	558	1.354 (1.347-1.361)
US minus 30 cities	764	959	782	533	1.226 (1.223-1.229)

^a^ The other race/ethnicity group includes all individuals other than those in the non-Hispanic Black or non-Hispanic White categories.

^b^ Data are for consolidated city and county.

**Figure 1.**
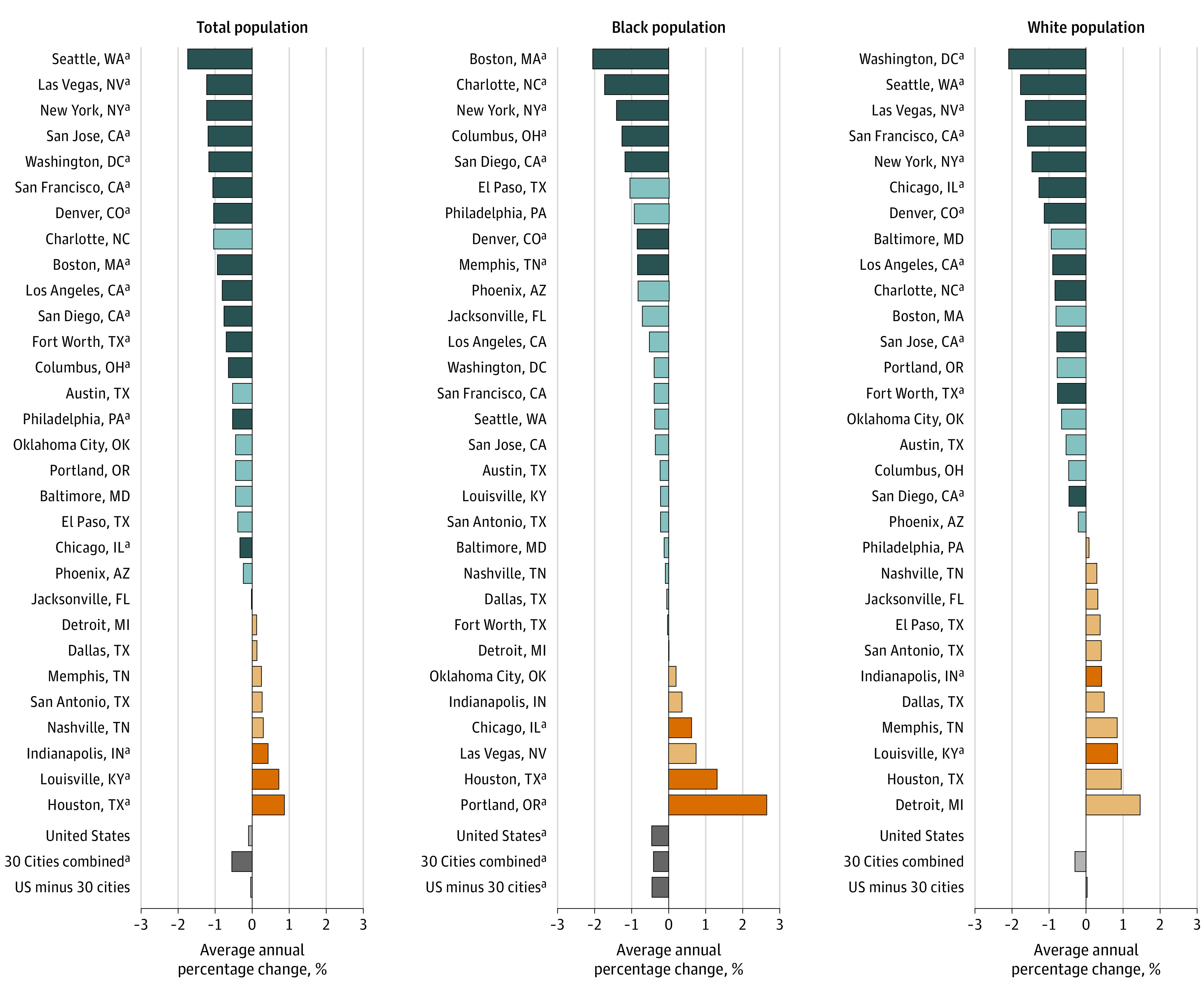
Average Annual Percentage Change in All-Cause Mortality Rates for the Total, Black, and White Populations, 2009 to 2018 Darker blue, orange, and gray bars indicate statistical significance. ^a^Significant changes, ie, *P* < .05.

Fourteen of the cities experienced significant declines in mortality between 2009 and 2018 (Figure 1). The average annual percentage change for these cities ranged from −0.33% in Chicago (95% CI, −0.51% to −0.14%; *P* = .003) to −1.74% (95% CI, −2.32% to −1.15%; *P* < .001) in Seattle. Three cities (Indianapolis, Louisville, and Houston) had significant increases in rates. The mortality rates for the remaining 13 cities were stable over time.

### Race-Specific Mortality Rates

The all-cause mortality rate among Black US residents was 960 per 100 000 individuals. Across cities, the rate among Black individuals ranged from 718 per 100 000 individuals (New York) to 1718 per 100 000 individuals (Las Vegas). The all-cause mortality rate among White US residents for the nation was 777 per 100 000 individuals. At the city level, rates among White individuals ranged from 428 per 100 000 individuals (Washington, DC) to 1462 per 100 000 individuals (Las Vegas). In all cities, the mortality rate for the other race/ethnicity category was lower than the rates among both Black and White populations.

Seven cities and the US showed a significant improvement in the average annual mortality rate among Black individuals between 2009 and 2018 (Figure 1). Mortality rates among Black individuals declined the most in Boston (average annual percentage change, −2.05%; 95% CI, −3.15% to −0.94%; *P* = .003) and Charlotte (average annual percentage change, −1.73%; 95% CI, −3.25% to −0.19%; *P* = .03). Conversely, 3 cities (Chicago, Houston, and Portland) experienced a significant increase in annual mortality rates among Black individuals. Portland’s increase was particularly large (average annual percentage change, 2.65%; 95% CI, 1.02% to 4.30%; *P* = .006). Twelve cities had significant declines in mortality rates among White individuals. Of these, Washington, DC, showed the largest improvements (average annual percentage change, −2.09%; 95% CI, −3.04% to −1.12%; *P* = .001). Indianapolis and Louisville had significant increases in mortality rates among White individuals. Eleven cities had consistent Black and White mortality rates over time.

### Racial Inequities

Racial inequities in mortality rates were assessed with rate ratios. In 2016 to 2018, the all-cause mortality rate among Black populations was 24% higher than among White populations in the United States (rate ratio = 1.236; 95% CI, 1.233-1.238). The rates among Black populations were significantly higher than those among White individuals in 29 of the 30 biggest cities (96.7%), with rate ratios ranging from 1.06 (95% CI, 1.03-1.09) in Jacksonville to 2.32 (95% CI, 2.22-2.42) in Washington, DC. In 1 city (El Paso), the mortality rates among Black and White populations were not significantly different (rate ratio = 1.05; 95% CI, 0.95-1.18). Overall, the racial inequities were greater in the 30 big cities than the United States as a whole.

**Figure 2.**
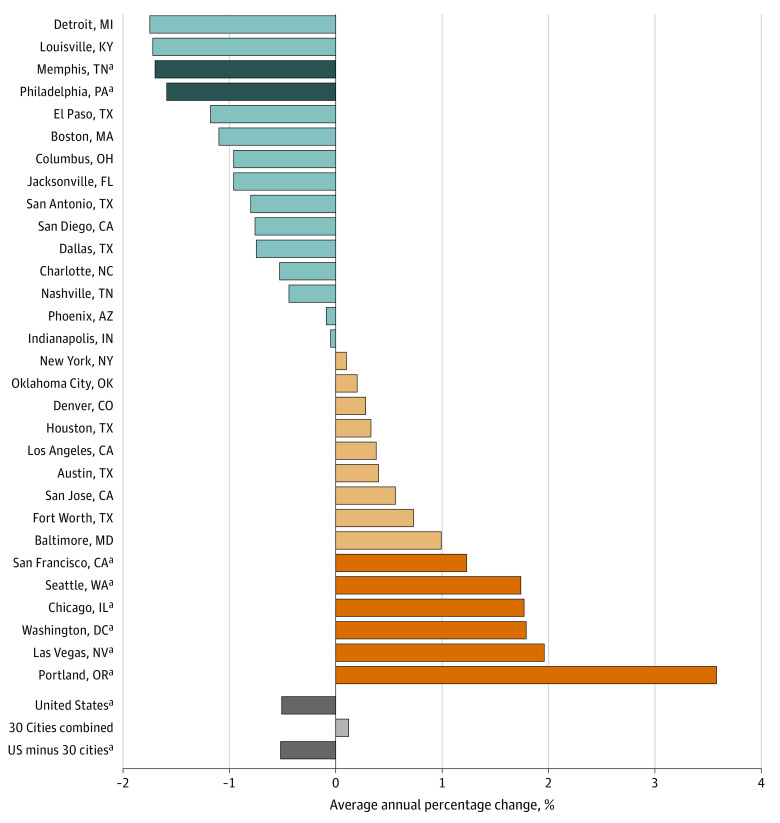
Change in Mortality Rate Ratios Between Black and White Populations, 2009 to 2018 Darker blue, orange, and gray bars indicate statistical significance. ^a^Significant changes, ie, *P* < .05.

Figure 2 shows that the US rate ratio between Black and White populations significantly decreased between 2009 and 2018 (average annual percentage change, −0.51%; 95% CI, −0.92% to −0.09%; *P* = .02). Two cities, Memphis and Philadelphia, also experienced significant annual decreases in rate ratios (average annual percentage change, −1.70%; 95% CI, −2.71% to −0.69%; *P* = .005; and −1.59%; 95% CI, −3.14% to −0.02%; *P* = .047, respectively). In contrast, 6 cities experienced significant increases in inequities (San Francisco; Seattle; Chicago; Washington, DC; Las Vegas; and Portland). Portland’s increase in racial inequities (associated with increases in the rate among Black individuals) was especially pronounced (average annual percentage change, 3.58%; 95% CI, 2.28% to 4.90%; *P* < .001).

**Figure 3.**
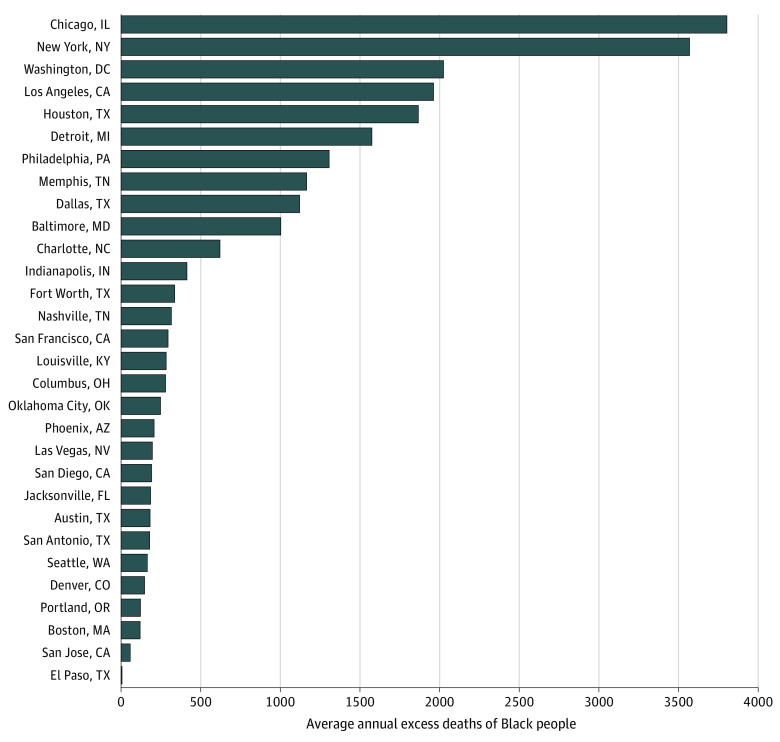
Average Annual Excess Black Deaths by City, 2016 to 2018 Excess deaths represent the average number of additional deaths in the Black population that occurred annually because of the higher mortality rate among Black vs White individuals.

In 2016 to 2018, 74 402 excess Black deaths occurred in the United States annually because the mortality rate among Black populations was higher than that among White populations. At the city level, the number of excess deaths was highest in Chicago and New York, with more than 3500 excess deaths each (Figure 3). In contrast, El Paso had only 6 excess Black deaths, and San Jose had fewer than 100.

### Comparing Cities by Outcome and Equity

**Figure 4.**
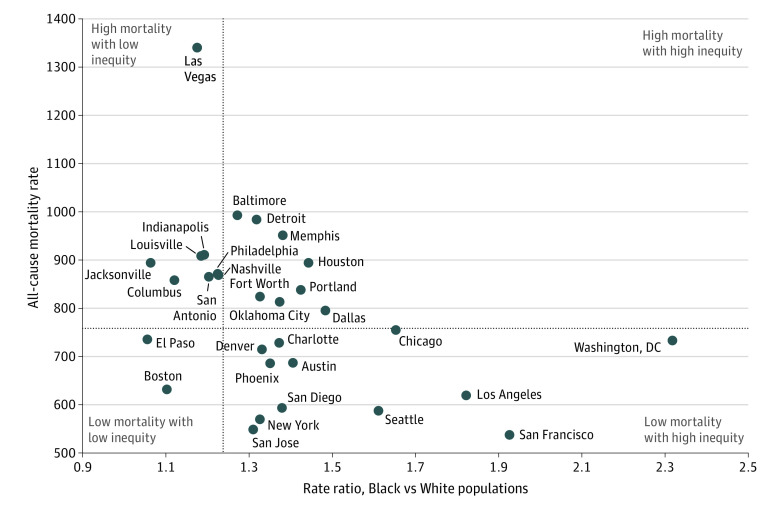
All-Cause Mortality Rates and Racial Inequities in Rates, 2016 to 2018 Quadrants are separated by lines representing the US mortality rate (horizontal line) and the US rate ratio between Black and White individuals (vertical line).

We plotted the 30 cities according to their rate ratio between Black and White individuals and their total all-cause mortality rate (Figure 4). The US mortality rate and the rate ratio between Black and White individuals were used to separate outcomes into quadrants. The lower-left quadrant, which includes Boston and El Paso, represents the best-performing cities. Conversely, the upper-right quadrant represents cities that had higher total mortality and racial inequity compared with the US overall. Eight cities were classified as worst-performing cities, including Memphis, Houston, and Portland. The bottom-right quadrant reveals that cities with the lowest total mortality rates often had the highest racial inequity. For example, San Francisco had the lowest all-cause mortality rate of the 30 cities; however, it had the second highest level of inequity (rate ratio = 1.92; 95% CI, 1.83-2.03). Conversely, Las Vegas and 7 other cities had mortality rates higher than that of the entire United States but lower racial inequity than the country as a whole.

## Discussion

This study addresses the increasing demand from cities and public health professionals for data that can be used to identify local issues, compare outcomes, and assess progress.^14,28^ We provided comprehensive city-level data, showing wide-ranging variations in all-cause mortality rates, racial inequities in rates, and changes over time in the 30 biggest US cities. This extends the analyses we present in *Unequal Cities: Structural Racism and the Death Gap in America’s 30 Largest Cities*,^20^ a book that examines racial inequities in mortality from the leading causes of death and explores the historical context and theoretic explanations for the entrenched inequities.

Consider that, in 2016 to 2018, Las Vegas had an all-cause mortality rate that was 2.5 times higher than that of San Francisco. To put the mortality rate in Las Vegas (1342 per 100 000 individuals) in context, it was greater than that of several countries in the global south, including Mexico (986 per 100 000 individuals) and Brazil (1001 per 100 000 individuals).^29^ A rate that high has not been observed for the United States as a whole since the 1950s.^1^ Similarly, Baltimore had levels of mortality similar to those observed in the United States more than 30 years ago.^1^ Only 14 cities experienced improvements in mortality rates during this period (2009-2018), whereas mortality rates in 3 cities worsened. The remaining 13 cities, and the United States as a whole, did not experience significant improvements in mortality rates during the decade, mirroring the much-discussed recent setback in life expectancy.^1,30,31,32^

The data also highlighted persistent racial inequities. The extent to which these inequities varied among cities warrants our attention. The disadvantage in mortality among Black individuals was relatively minor (or even nonexistent) in some cities but was substantial in others. For instance, in Washington, DC, the rate among Black individuals was 2.3 times the rate among White individuals. In both Chicago and New York, more than 3500 Black people died annually because of this health inequity. Racial inequities worsened in 6 cities and improved in only 2.

Although an empirical exploration of the factors associated with such variation in mortality rates and inequities in rates between cities is beyond the scope of the current study, we can consider potential explanations. Previous analyses have shown that city-level characteristics associated with all-cause mortality include poverty, median household income, percentage of Black residents, racial segregation, and income inequality.^20,33,34^ However, all of these factors must be interpreted with historical context; they are recognized as social determinants of health, but they are also the products of even more deep-rooted fundamental causes. An increasing body of work in social epidemiology has attempted to disentangle population-level social determinants of health from structural determinants of health inequities, recognizing, for example, that racial composition is a proxy for a wide range of social, political, and economic processes that have shaped communities across the country.^35^ Likewise, data on income inequality reflect decades’ worth of economic processes associated with the declining power of labor unions, deindustrialization, and gentrification. It is plausible that city-level variability in racial inequities in mortality reflects differential exposures to policies and systems that create and reinforce this wide range of social drivers of health inequities.

Potential reasons for improvements in mortality rates in approximately half of the cities could include similar factors, such as racial composition, per-capita income, and population density.^36^ Recent research at the state level found associations between changes in life expectancy and policies related to civil rights, criminal justice, education, environment, housing, and health care, among other domains.^37^ Geographically, mortality trajectories at the county level have been found to vary by region, with negative trajectories being concentrated in the Southeast.^30^ Research on population-level changes in cause-specific mortality can also suggest more specific avenues for cities.^38^

Most big cities (22 of 30) did not observe any statistically significant changes in levels of racial inequities. This is remarkably disappointing, given national and local efforts focused on health equity. Simultaneously, these results may serve to reinforce the need for systems-level change, shifting community health improvement efforts from behavioral to structural interventions.

These types of comparative data can increase awareness and advocacy for equity, guide the allocation of scarce resources to the appropriate locations or population subgroups, and highlight cities that might be implementing effective population health strategies.^39,40,41,42^ However, actions undertaken to improve the health of the overall population often differ from those required to improve health equity.^43^ Cities that would like to focus on improving overall mortality have a plethora of resources offering guidance and examples.^44^ For instance, the Community Health Improvement Navigator database provides tools for multisector, collaborative health initiatives.^45^ Resources from the Community Guide,^46^ County Health Rankings,^3^ and the BUILD Health Challenge^47^ also offer extensive insight and tools for communities.

In contrast, actions needed to improve health equity should specifically address the inequitable social conditions that sustain the disparities.^43^ A 2019 review of effective initiatives for achieving equity^43^ included efforts related to early childhood development, child poverty, job opportunities, and environmental conditions in disadvantaged communities. In sum, reducing inequities in health requires addressing deep-rooted structural racism in US society.^48^ Existing sources of health-related data at the neighborhood or census tract level can further help cities target efforts to the geographic areas needing the most support. In addition, race equity tools can help cities evaluate policies for their influence on inequities.^49^

City-level data are critical because policy change is most likely to occur at the city level, not the neighborhood or census tract level. However, conducting this type of analysis is time consuming and complex; thus, local health departments often rely on data not specific to their actual population.^50^

Understanding all-cause mortality patterns using population-based data is an important first step. To build on this, data on city-level mortality rates (and inequities within) for the leading causes of death in the United States are needed to further our understanding of mortality disparities and to better support city-level efforts. Ecologic studies linking these findings with city-level demographic and socioeconomic characteristics will also provide more insight.^20,51^ It may also be instructive to specifically examine cities conspicuous in our analyses for significant improvements in overall mortality or in health equity. For example, what might explain the substantial improvements in equity in Memphis and Philadelphia? Do they have city or county health plans that focus on equity? Have major academic, health care, or community organizations played a role? Or do demographic characteristics help to explain their success? Focusing on modifiable factors associated with improving (or worsening) mortality rates could help to inform future policy and programmatic efforts.

More broadly, we want to reiterate calls for information on equity to be provided as part of all major sources of health information. To date, few sources of mortality data provide this. The Health Disparities widget, part of the Healthy People website,^19^ is a notable exception. Without a systematic (and public) documentation of racial inequities at any level, they are easier to overlook or ignore.^52^

### Limitations

This work has several limitations. First, race and ethnicity data from death certificates may be inaccurate because they are based on proxy interviews or observations. However, research suggests race reporting is highly accurate for White and Black races and Hispanic ethnicity.^53,54^ Our estimate of the non-Hispanic Black population assumes the proportion of the Black population that is non-Hispanic has remained static since the 2010 census. If the Hispanic Black population has increased in any of our analyzed geographies, the true non-Hispanic Black population would be smaller than we estimated, and our outcomes would have therefore underestimated the true mortality rate. We also recognize the complexity of quantifying inequities and acknowledge that we included several, but not all, possible measures (because of the number of outcomes and populations).^24^ Our inequities analyses are limited to examining differences between Black and White populations. We did not include the other largest race/ethnicity groups for several reasons. In the US, Black and White populations are frequently used in public health as representative of the extremes of privilege and marginalization.^55,56^ Furthermore, Hispanic/Latinx and Asian populations generally have significantly lower mortality rates than both Black and White populations.^1^ In addition, the smaller number of deaths for these groups would limit the number of cities we could include in the analyses. Additionally, the current analyses did not include an examination of ecologic factors and thus cannot account for demographic or socioeconomic changes that may have occurred within the cities during the study period.

## Conclusions

The findings of this study suggest that mortality in the United States is associated with one’s skin color and city of residence. This reflects the complex dynamics connecting structural racism, demography, public policy, health care, and the lived experience of communities and individuals. To our knowledge, we have provided the first comprehensive summary of all-cause mortality rates and related racial inequities at the city level. These data will help cities more strategically pursue specific policy and programmatic changes to improve health and health equity for their residents. Given that 4 of 5 US individuals currently live in urban areas (and this number continues to increase),^57,58^ these actions are needed to enable the United States to move toward its goals of increasing healthy life expectancy and eliminating health inequities.

## Notes

Supplement. **eTable 1.** Selected City-Level Sociodemographic Characteristics for the US and the 30 Largest US Cities **eTable 2.** Age-Adjusted All-Cause Mortality Rates and Measures of Disparities Over 3 Time Periods (2010-2012, 2013-2015, and 2016-2018)
