# Supplementary material for: Comparison of All-Cause Mortality Rates and Inequities Between Black and White Populations Across the 30 Most Populous US Cities
Source: JAMA Netw Open. 2021 Jan 20;4(1):e2032086. doi: 10.1001/jamanetworkopen.2020.32086 (PMC9386890; doi:10.1001/jamanetworkopen.2020.32086)
Supplement: Supplement. — eTable 1. Selected City-Level Sociodemographic Characteristics for the US and the 30 Largest US Cities eTable 2. Age-Adjusted All-Cause Mortality Rates and Measures of Disparities Over 3 Time Periods (2010-2012, 2013-2015, and 2016-2018) [file jamanetwopen-e2032086-s001.pdf]

Supplemental Online Content

Benjamins MR, Silva A, Saiyed NS, De Maio FG. Comparison of all-cause mortality rates and inequities between Black and White populations across the 30 most populous US cities. *JAMA Netw Open*. 2021;4(12):e2032086. doi:10.1001/jamanetworkopen.2020.32086

eTable 1. Selected City-Level Sociodemographic Characteristics for the US and the 30 Largest US Cities

eTable 2. Age-Adjusted All-Cause Mortality Rates and Measures of Disparities Over 3 Time Periods (2010-2012, 2013-2015, and 2016-2018)

This supplemental material has been provided by the authors to give readers additional information about their work.

© 2021 Benjamins MR et al. *JAMA Network Open.*

eTable 1. Selected City-Level Sociodemographic Characteristics for the US and the 30 Largest US Cities

City, State	Population	Percent NH White	Percent NH Black	Percent Other[1]	Percent ≥65 Years	Median Age (in years)	Percent Male
United States	*318,558,162*	*61*	*12*	*27*	*15*	*38*	*49*
Austin, TX	907,779	49	7	44	8	32	51
Baltimore, MD	621,000	28	63	9	12	35	47
Boston, MA	658,279	45	23	32	11	32	48
Charlotte, NC	808,834	43	35	22	10	34	48
Chicago, IL	2,714,017	32	31	37	11	34	49
Columbus, OH	837,038	58	28	14	9	32	49
Dallas, TX	1,278,433	29	24	47	10	33	50
Denver, CO	663,303	53	9	38	11	34	50
Detroit, MI	683,443	10	79	11	13	35	47
El Paso, TX	678,058	14	3	83	12	33	49
Fort Worth, TX	815,930	41	19	40	9	32	49
Houston, TX	2,240,582	25	22	53	10	33	50
Indianapolis, IN	846,674	57	28	15	11	34	48
Jacksonville, FL	856,616	53	30	17	13	36	48
Las Vegas, NV	613,295	46	11	43	14	37	50
Los Angeles, CA	3,918,872	29	9	62	12	35	50
Louisville, KY	611,573	67	23	10	14	37	48
Memphis, TN	655,857	27	63	10	11	34	48
Nashville, TN	643,771	56	28	16	11	34	48
New York, NY	8,461,961	32	22	46	13	36	48
Oklahoma City, OK	620,015	55	14	31	12	34	49
Philadelphia, PA	1,559,938	35	42	23	13	34	47
Phoenix, AZ	1,555,324	44	7	49	10	33	50
Portland, OR	620,589	72	6	22	12	37	49
San Antonio, TX	1,439,358	26	7	67	11	33	49
San Diego, CA	1,374,812	43	6	51	12	34	50
San Francisco, CA	850,282	41	5	54	14	38	51
San Jose, CA	1,009,363	27	3	70	11	36	50
Seattle, WA	668,849	66	7	27	12	36	50
Washington, DC	659,009	36	47	17	11	34	47

Abbreviations: NH, non-Hispanic; HS, high school
Source: American Community Survey, Demographic and Housing Estimates, 2016. Downloaded from https://data.census.gov/cedsci/r, Table DP05.
[1]"Other" category includes all individuals not classified as NH Black or NH White. It was calculated by subtracting the Black and White population estimates from the total population estimates for the U.S. and each city.

© 2021 Benjamins MR et al. *JAMA Network Open.*

eTable 2. Age-Adjusted All-Cause Mortality Rates and Measures of Inequities Over 3 Time Periods (2010-2012, 2013-2015, and 2016-2018)							
	2010-2012						
**Location**	**Total Mortality Rate**	**NH Black Mortality Rate**	**NH White Mortality Rate**	**Other Mortality Rate**	**B:W Rate Ratio**	**Rate Ratio CI**	**Excess Black Deaths**
United States	769	972	778	556	1.25	(1.247-1.252)	67257
Austin, TX	740	997	735	660	1.36	(1.28-1.44)	141
Baltimore, MD	1018	1114	929	357	1.20	(1.16-1.24)	735
Boston, MA	686	827	733	391	1.13	(1.08-1.18)	133
Charlotte, NC	757	975	694	475	1.40	(1.35-1.46)	498
Chicago, IL	780	1027	693	540	1.48	(1.46-1.51)	3010
Columbus, OH	900	937	918	411	1.02	(0.99-1.06)	106
Dallas, TX	802	1053	770	542	1.37	(1.33-1.41)	800
Denver, CO	770	974	740	759	1.32	(1.25-1.39)	139
Detroit, MI	1012	1055	957	601	1.10	(1.06-1.15)	583
El Paso, TX	768	921	841	741	1.10	(0.98-1.22)	17
Fort Worth, TX	869	1105	877	645	1.26	(1.21-1.31)	248
Houston, TX	860	1157	812	698	1.43	(1.40-1.46)	1560
Indianapolis, IN	895	1028	886	322	1.16	(1.13-1.20)	303
Jacksonville, FL	900	1032	890	549	1.16	(1.12-1.20)	310
Las Vegas, NV	1422	1660	1550	979	1.07	(1.03-1.12)	64
Los Angeles, CA	647	1113	636	530	1.75	(1.71-1.78)	1781
Louisville, KY	880	1046	872	302	1.20	(1.16-1.24)	232
Memphis, TN	948	1139	768	324	1.48	(1.44-1.53)	1200
Nashville, TN	866	1039	851	348	1.22	(1.17-1.27)	251
New York, NY	633	784	601	562	1.31	(1.29-1.32)	3392
Oklahoma City, OK	844	1079	850	643	1.27	(1.21-1.33)	158
Philadelphia, PA	918	1060	857	648	1.24	(1.21-1.26)	1303
Phoenix, AZ	734	951	742	659	1.28	(1.21-1.35)	139
Portland, OR	874	1048	900	579	1.16	(1.09-1.25)	51
San Antonio, TX	877	1080	912	825	1.18	(1.13-1.24)	140
San Diego, CA	618	899	635	531	1.42	(1.35-1.49)	199
San Francisco, CA	591	1130	647	481	1.75	(1.66-1.83)	280
San Jose, CA	599	896	700	494	1.28	(1.17-1.40)	46
Seattle, WA	677	989	685	517	1.44	(1.36-1.53)	140
Washington, DC	792	1007	504	426	2.00	(1.92-2.08)	1703
30 Cities Combined	*762*	*990*	*752*	*587*	*1.32*	*(1.311-1.324)*	*19744*
US Minus 30 Cities	*770*	*968*	*780*	*547*	*1.24*	*(1.237-1.244)*	*48642*

© 2021 Benjamins MR et al. *JAMA Network Open.*

eTable 2. (continued)							
	2013-2015						
**Location**	**Total Mortality Rate**	**NH Black Mortality Rate**	**NH White Mortality Rate**	**Other Mortality Rate**	**B:W Rate Ratio**	**Rate Ratio CI**	**Excess Black Deaths**
United States	761	953	774	548	1.23	(1.228-1.234)	67392
Austin, TX	721	982	723	636	1.36	(1.29-1.44)	156
Baltimore, MD	1081	1137	1048	445	1.08	(1.05-1.12)	417
Boston, MA	660	736	726	405	1.01	(0.97-1.06)	29
Charlotte, NC	731	948	665	489	1.42	(1.37-1.48)	578
Chicago, IL	765	1024	676	538	1.52	(1.49-1.54)	3144
Columbus, OH	883	946	901	371	1.05	(1.02-1.09)	136
Dallas, TX	819	1094	777	580	1.41	(1.37-1.45)	936
Denver, CO	749	965	729	696	1.32	(1.26-1.39)	145
Detroit, MI	949	991	912	519	1.09	(1.04-1.14)	518
El Paso, TX	744	780	821	722	0.95	(0.85-1.06)	-2
Fort Worth, TX	853	1077	884	576	1.22	(1.17-1.27)	244
Houston, TX	905	1189	863	770	1.38	(1.35-1.41)	1578
Indianapolis, IN	898	1050	887	393	1.18	(1.15-1.22)	354
Jacksonville, FL	875	997	877	518	1.14	(1.10-1.17)	282
Las Vegas, NV	1403	1656	1536	994	1.08	(1.04-1.12)	73
Los Angeles, CA	625	1095	621	516	1.76	(1.73-1.80)	1804
Louisville, KY	883	1044	875	378	1.19	(1.15-1.24)	253
Memphis, TN	962	1117	795	525	1.40	(1.36-1.45)	1135
Nashville, TN	865	1051	843	514	1.25	(1.20-1.29)	284
New York, NY	608	746	583	540	1.28	(1.26-1.29)	3180
Oklahoma City, OK	841	1115	852	532	1.31	(1.25-1.37)	208
Philadelphia, PA	877	1000	830	658	1.20	(1.18-1.23)	1134
Phoenix, AZ	711	884	717	661	1.23	(1.17-1.30)	135
Portland, OR	879	1175	901	601	1.30	(1.22-1.39)	87
San Antonio, TX	880	1093	907	832	1.21	(1.16-1.26)	169
San Diego, CA	605	878	635	510	1.38	(1.32-1.45)	194
San Francisco, CA	559	1086	599	468	1.82	(1.73-1.91)	284
San Jose, CA	565	917	662	471	1.39	(1.27-1.51)	64
Seattle, WA	651	1025	652	516	1.57	(1.48-1.67)	168
Washington, DC	767	983	484	468	2.03	(1.95-2.12)	1764
30 Cities Combined	*747*	*969*	*743*	*579*	*1.30*	*(1.297-1.310)*	*19579*
US Minus 30 Cities	*763*	*950*	*777*	*540*	*1.22*	*(1.220-1.226)*	*49379*

© 2021 Benjamins MR et al. *JAMA Network Open.*

eTable 2. (continued)							
	2016-2018						
Location	Total Mortality Rate	NH Black Mortality Rate	NH White Mortality Rate	Other Mortality Rate	B:W Rate Ratio	Rate Ratio CI	Excess Black Deaths
United States	759	960	777	539	1.24	(1.233-1.238)	74402
Austin, TX	687	980	698	563	1.40	(1.33-1.48)	181
Baltimore, MD	993	1107	870	343	1.27	(1.23-1.31)	1003
Boston, MA	632	735	666	450	1.10	(1.06-1.15)	119
Charlotte, NC	729	931	678	385	1.37	(1.33-1.42)	622
Chicago, IL	756	1065	644	523	1.65	(1.62-1.68)	3804
Columbus, OH	858	975	870	323	1.12	(1.08-1.16)	279
Dallas, TX	795	1092	736	590	1.48	(1.44-1.52)	1121
Denver, CO	715	918	689	683	1.33	(1.27-1.40)	147
Detroit, MI	984	1048	795	670	1.32	(1.26-1.38)	1575
El Paso, TX	735	894	847	704	1.05	(0.95-1.18)	6
Fort Worth, TX	823	1103	831	588	1.33	(1.28-1.38)	336
Houston, TX	895	1226	850	734	1.44	(1.41-1.47)	1866
Indianapolis, IN	911	1068	896	451	1.19	(1.16-1.23)	413
Jacksonville, FL	894	985	926	499	1.06	(1.03-1.09)	185
Las Vegas, NV	1342	1718	1462	924	1.18	(1.13-1.22)	196
Los Angeles, CA	619	1102	605	522	1.82	(1.78-1.86)	1962
Louisville, KY	910	1069	902	425	1.19	(1.15-1.23)	283
Memphis, TN	951	1086	786	475	1.38	(1.34-1.43)	1166
Nashville, TN	868	1041	849	470	1.23	(1.18-1.27)	317
New York, NY	570	718	542	504	1.33	(1.31-1.34)	3569
Oklahoma City, OK	813	1114	811	535	1.37	(1.31-1.44)	247
Philadelphia, PA	870	1013	827	597	1.22	(1.20-1.25)	1307
Phoenix, AZ	686	938	694	612	1.35	(1.29-1.42)	207
Portland, OR	837	1209	849	618	1.42	(1.34-1.52)	121
San Antonio, TX	866	1105	918	802	1.20	(1.16-1.25)	178
San Diego, CA	592	857	622	499	1.38	(1.32-1.45)	192
San Francisco, CA	537	1102	573	455	1.92	(1.83-2.03)	294
San Jose, CA	549	856	654	455	1.31	(1.20-1.42)	58
Seattle, WA	588	945	587	484	1.61	(1.52-1.71)	165
Washington, DC	733	993	428	402	2.32	(2.22-2.42)	2024
30 Cities Combined	*724*	*971*	*717*	*558*	*1.35*	*(1.347-1.361)*	*23064*
US Minus 30 Cities	*764*	*959*	*782*	*533*	*1.23*	*(1.223-1.229)*	*54827*

Abbreviations: NH, non-Hispanic; B:W, Black:White; CI, confidence interval

Notes: Mortality rates are shown per 100,000 population. The "Other" race/ethnic group includes all individuals other than those in the NH Black or NH White categories. Excess deaths represent the average number of additional Black deaths that occurred annually due to the higher Black mortality rate compared to the White rate. It is a weighted calculation, so the numbers for each city will not add up to the total for 30 cities combined, for example.

© 2021 Benjamins MR et al. *JAMA Network Open.*
